# Imaging investigation of cervicocranial artery dissection by using high resolution magnetic resonance VWI and MRA: qualitative and quantitative analysis at different stages

**DOI:** 10.1186/s12880-023-01133-z

**Published:** 2023-11-13

**Authors:** Weiqiong Ma, Kexin Zhou, Bowen Lan, Kangyin Chen, Wuming Li, Guihua Jiang

**Affiliations:** 1 The Second School of Clinical Medicine, Southern Medical University, NO.1023 North Road of Shatai, Baiyun District, Guangzhou, Guangdong China; 2 Department of Medical Imaging, Guangdong Second Provincial General Hospital, NO.1 Road of Shiliugang, Haizhu District, Guangzhou, Guangdong China; 3 Department of Radiology, Huizhou Central People’s Hospital, NO.41 North Road of Eling, Huicheng District, Huizhou, Guangdong China

**Keywords:** Cervicocranial artery dissection, High resolution magnetic resonance vessel wall imagin, Magnetic resonance angiography, Different stages

## Abstract

**Background:**

To explore the value of magnetic resonance angiography (MRA) and high resolution magnetic resonance vessel wall imaging (HRMR-VWI) in cervicocranial artery dissection (CCAD) for the disease diagnosis, course staging and treatment. On the basis of qualitative evaluation, this study also extract the changes of different stages in vessel wall in different vessel segments to identify imaging indicators for the quantitative evaluation of CCAD.

**Methods:**

We retrospectively enrolled 34 patients with CCAD (38branches) with conventional MRA and HRMR-VWI examinations. Two radiologists independently analyzed imaging features of vessel wall and lumen in the different stages, and the typical sign detection of artery dissection were compared between MRA and HRMR-VWI. Then the parameters of vessel wall was quantitatively evaluated by the post-processing software (Vesselmass, Leiden University Medical Center, Leiden, The Netherlands.

**Results:**

HRMR-VWI revealed typical sign detection of artery dissection in all patients in the acute and subacute stage. Among them, the intimal flap/double lumen sign ditection were more common than the MRA, there was significant difference (*P* = 0.012). MRA revealed typical sign detection of artery dissection in more than half the patients, and the detection was no significant difference at the chronic stage between MRA and HRMR-VWI (*P* = 1.000/1.000/0.761). In the acute and subacute stage, the typical sign detection of intramural hematoma and Grade II enhancement revealed by HR-MRI was higher than the observations in the chronic stage (*P* = 0.000/0.000/0.016), while there was no significant difference by MRA (*P* = 0.902). The values of wall thickness, relative signal intensity of vessel wall enhancement, relative signal intensity of intramural hematoma (IMH), and percentage of stenosis in CCAD decreased from acute to subacute and then to chronic stages. Each quantitative parameter in patients with CCAD in the early stages (i.e., acute and subacute stages) was significantly different from that in patients with CCAD in the recovered group at chronic stage (*P* < 0.05). Wall thickness and relative signal intensity of vessel wall enhancement in patients with CCAD in the early stages were not significantly different from those in patients with CCAD in the incompletely recovered group at chronic stage (*P* > 0.05).

**Conclusions:**

As the only noninvasive imaging technology, HRMR-VWI displays the structure of the vessel wall in vivo, showing not only excellent performance in the early diagnosis of CCAD, but also describing the changes of different stages in the qualitative and quantitative characteristics of vessel wall. It also helps to guide the diseasediagnosis, course staging and treatment of CCAD. Although the diagnostic efficacy of MRA was not as good as HRMR-VWI, it should be the first choice of method for routine examination in evaluating CCAD, especially at the chronic stage of CCAD.

## Background

Cervicocranial artery dissection (CCAD) is a common cause of stroke in young and middle-aged adults. Cases of intracranial artery dissection (ICAD) have mainly been reported in Asia, while cases of carotid artery dissection have mainly been reported in Europe [1–3]. CCAD may lead to severe cerebral ischemia or cerebral hemorrhage, with high morbidity and mortality rates. Early diagnosis of CCAD is particularly important; however, CCAD diagnosis has been challenging, mainly because of the unclear risk factors and pathogenesis and the lack of specific symptoms and signs. Diagnosis mostly relies on imaging. However, the diagnostic imaging criteria are unclear and the dynamic geometric changes in CCAD during the healing process may obscure the imaging manifestations [4, 5], thereby complicating the accuracy of diagnostic evaluation and challenging the differentiation from other vascular diseases.

Digital subtraction angiography (DSA) was previously considered the gold standard for CCAD diagnosis. However, as an invasive procedure, DSA is associated with a risk of complications [6]. In addition, because of the lack of cross-sectional images, DSA has low detection rates for the direct signs of aortic dissection (i.e., intimal flap and double lumen), which may lead to false-negative diagnosis. In recent years, high-resolution magnetic resonance vessel wall imaging (HRMR-VWI) has started to be used in CCAD. HRMR-VWI is a noninvasive and reproducible method with a high resolution. It not only provides information on the lumen, but also visualizes vessel walls, thereby effectively characterizing the direct signs, such as intimal flap, double lumen, and intramural hematoma (IMH). Hence, HRMR-VWI may be superior to DSA in diagnosing CCAD [6, 7].

Currently, optimum treatment for patients with CCAD is unknown. The preferred and widespread practice is surgical or endovascular treatment in patients with CCAD with subarachnoid haemorrhage and mass effffect, whereas patients with CCAD without subarachnoid haemorrhage and cerebral ischaemia tend to be given medical treatment. Therefore, different progression and underlying pathological mechanisms can lead to different therapeutic schedule [8, 9]. And some studies have shown that HRMR-VWI reveals the pathological changes in CCAD during the healing process, which may help to provide guidance for the diagnosis and staging of CCAD to guide subsequent treatmen [10, 11]. A study by Heldner et al. [12] indicated certain variation patterns of imaging signal of intramural hematoma. In general, T1-weighted images of IMH show high-intensity signal 4 to 40 days after disease onset, while T2-weighted images (T2WIs) show high-intensity signal 8 days after disease onset. The signal intensity becomes low approximately a month after disease onset. In approximately 80% of the patients, IMH are no longer detectable three months after disease onset. Nearly IMH are absorbed by six months after disease onset in nearly all patients. Jung et al. followed up the imaging features of spontaneous CCAD in the chronic phase and showed that HRMR-VWI detected vessel wall thickening accompanied with enhancement even in cases of complete recovery revealed by DSA [4]. During the healing process, compensatory intimal thickening occurs around the pseudo-lumen [13], which is consistent with the wall changes. This indicates that HRMR-VWI may noninvasively display the changes in vessel wall by revealing the histopathological features of CCAD. However, the above studies have mainly focused on the qualitative diagnosis of the dynamic changes in certain imaging features, without fully showing the relationship between the dynamic changes in HRMR-VWI and the disease staging and prognosis. In addition, the qualitative diagnosis relies too much on the experience of radiologists and physicians; thus, it is highly subjective, and its accuracy is questionable.

Moreover, HRMR-VWI requires specific hardware and technical implementation [14] and thus cannot be used as routine examination of CCAD, especially in patients with artery dissection but no apparent stroke symptoms. Magnetic resonance angiography (MRA) is a routine examination method for intracranial arterial assessment. However, few studies have evaluated the diagnostic value of MRA for CCAD at different stages. Hence, this study aimed to explore the value of MRA and HRMR-VWI in CCAD diagnosis and disease staging by comparing the imaging signs of the vessel lumen and wall of CCAD at different stages. On the basis of qualitative evaluation, this study also used post-processing software (VesselMass, Leiden University Medical Center, Leiden, The Netherlands) to extract the changes in vessel wall in different vessel segments to identify imaging indicators for the quantitative evaluation of CCAD to provide more comprehensive, objective, and accurate guidance for the diagnosis and staging of CCAD and to facilitate understanding of the progression and underlying pathological mechanisms of CCAD to guide subsequent treatment.

## Methods

### Patients

This retrospective study was approved by our Institutional Review Board, and the need for informed consent from the patients was waived as the patients’ information was anonymized and de-identified before the evaluation. Among the 454 patients who underwent 3.0T HRMR-VWI and MRA in our hospital from September 2018 to December 2020. Thirty-eight diseased blood vessels in 34 patients were included, according to the following criteria: (1) diagnosis of CCAD was based on the clinical symptoms and DSA manifestations; (2) the lumen and wall imaging of the head and neck region were performed simultaneously after symptom onset; and (3) without receiving any endovascular or surgical procedures before HRMR-VWI. The exclusion criteria of this study were patients with other cerebrovascular diseases (e.g., moyamoya disease, vasculitis, fibromuscular dysplasia, and identifiable and unstable plaque accompanied with ulcer) and cardiogenic embolism. The clinical characteristics, including sex, age, risk factors (e.g., high blood pressure, hyperlipidemia, diabetes, history of trauma, history of recent infection, history of alcohol consumption, smoking habit, and body mass index), symptoms, time interval from the onset of symptoms to HRMR-VWI imaging, segmentation stages based on symptoms, and vessel segments of the lesion of each patient were recorded.

### Image acquisition

All patients underwent MRA and HRMR-VWI using 3T MRI Scanners (Magnetom Verio, Magnetom Skyra, and Magnetom Prisma, Siemens, Erlangen, Germany) and the 64-Channel Head/Neck coil. The scanning plans included the T2 weighted image (T2WI), DWI, apparent diffusion coefficient (ADC), time-of-flight (TOF)-MRA, three-dimensional T1 (3D-T1)-weighted black-blood, contrast-enhanced (CE)-MRA, and CE-3D-T1-weighted black-blood. The 3D-T1-weighted black blood MRI was performed using Siemens’ DANTE-SPACE technology, using the following specific parameters: the repetition time/the echo time = 850/21 ms, field of view = 205 mm, matrix size = 384 × 384, layer number = 256, layer thickness = 0.53 mm, voxel size = 0.53 × 0.53 × 0.53 mm, and collection time = 8 min. The parameters for the DANTE preparation include: flip angle = 8°, phase increment = 0°, number of pulses = 150, interpulse repeat time = 1.5 ms, Gxyz = 20 mT/m. For T2WI the parameters were as follows: the repetition time/the echo time = 4500/99 ms, field of view = 220 mm, matrix size = 384 × 288, layer number = 20, layer thickness = 5.0 mm, voxel size = 0.3 × 0.3 × 5.0 mm. For DWI the parameters were as follows: the repetition time/the echo time = 3010/60 ms, field of view = 220 mm, matrix size = 192 × 192, layer number = 20, layer thickness = 5.0 mm, voxel size = 1.1 × 1.1 × 5.0 mm. For TOF-MRA the parameters were as follows: the repetition time/the echo time = 20/3.69 ms, field of view = 220 mm, matrix size = 384 × 268.8, layer number = 320, layer thickness = 0.7 mm, voxel size = 0.6 × 0.6 × 0.7 mm. For CE-MRA the parameters were as follows: the repetition time/the echo time = 3.73/1.31 ms, field of view = 350 mm, matrix size = 384 × 299.5, layer number = 320, layer thickness = 1.0 mm, voxel size = 0.8 × 0.8 × 1.0 mm. The CE-3D-T1-weighted MRI scan started 2 min after injection of 0.1 mmol/kg contrast agent (MultiHance, Bracco SpA, Milano, Italy) with an injection rate of 0.1 ml/s.

The reasons for using both TOF-MRA and CE-MRA to evaluate the vessel lumen were as follows: the intracranial artery has a fixed position, which is minimally affected by respiration and vascular pulsation, making it suitable for TOF-MRA, which has high resolution. However, the vessels in the neck are long and are greatly affected by respiration, swallowing, and vascular pulsation, making them suitable for CE-MRA.

### Image analysis

**Fig. 1:**
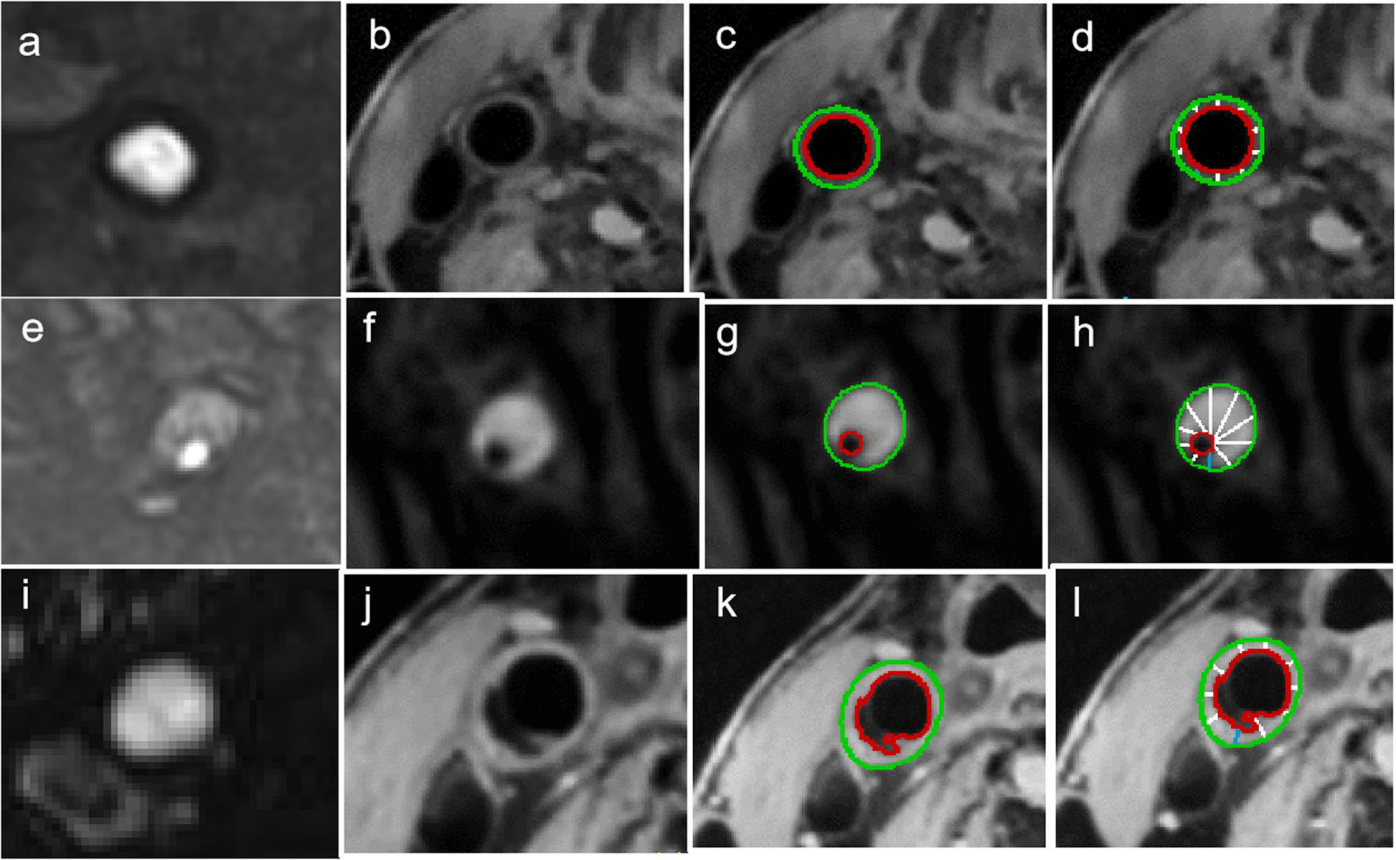
Outlines of the external and internal lumen contours in the 2D cross-sectional images using the vessel mass software. The entire vessel wall area was automatically divided into ten evenly spaced segments by two contours. **a**-**d** The normal vessel wall contour; **e**-**h** the intramural hematoma (IMH) contour; and **i**-**l** the double-lumen contour

Two radiologists with 5 and 8 years of experience in neuroimaging interpreted the MRA and HRMR-VWI images on the post-processing workstation. The radiologists were unaware of the DWI and clinical information of the patients. Inconsistent results of image analysis were further discussed or consulted with a third radiologist with 20 years of experience in neuroimaging until an agreement was reached. The imaging manifestations of MRA included double lumen, irregular vessel wall direction, aneurysmal dilatation or fusiform dilatation, and tapered stenosis or occlusion. The imaging manifestations of HRMR-VWI included intimal flap, double lumen, aneurysmal protrusion, grading of vessel wall enhancement, and IMH. According to the time intervals from the onset of symptoms to HRMR-VWI imaging, the disease was divided into three stages, namely acute stage (0–7 days), subacute stage (7–60 days), and chronic stage (> 60 days). According to clinical and imaging findings, patients at the chronic stage were divided into a recovered group and an incompletely recovered group. The recovered group referred to patients with normal vessel lumen and wall imaging morphology, and patients with normal lumen imaging morphology but limited vessel wall thickening and mild vessel wall abnormalities (see yellow arrows in Fig. 4). The incomplete recovered group referred to patients with morphological changes in the vessel lumen and wall imaging (see red arrows in Figs. 4 and 5). The intimal flap was defined as a linear partition that passed through the flow cavity and extended to the side wall on the continuous cross-section of each MRI sequence [15] (yellow arrows in Figs. 3 and 5). A double lumen was defined when the blood flow was detected in both the false lumen and the true lumen [16] (red arrows in Figs. 4 and 5). Aneurysmal protrusion, including dissecting aneurysm and pseudoaneurysms, was defined as an aneurysm with a vessel diameter exceeding the diameter of the normal distal artery by ≥ 1.5-fold [6] (Fig. 1, green arrows in Figs. 5 and 6). IMH was defined as a false lumen filled with hematoma without blood flow, showing iso- or hypersignal shadow on images before angiography [17, 18] (red arrows in Fig. 3). The degree of wall enhancement of the blood vessels was divided into 3 levels as follows: Level 0, similar to a normal blood vessel wall; Level 1, greater than Level 0 but less than venous wall enhancement; Level 2, similar to or greater than venous wall enhancement.

The dissected vessel segment was covered with two-dimensional (2D) continuous cross-sections, and 10 slices each from the proximal and distal ends of the dissected vessel segment were taken as normal group data. All cross-sectional images were then transferred to a commercial software (VesselMass, Medical Center, University of Leiden, Leiden, Netherlands), followed by manual drawing of two contours (external and internal lumen contours) along the interface between the lumen and vessel wall, and between the vessel wall and surrounding tissues. The contours were completed to maintain the continuity of the curvature of the blood vessel when the margin was partially invisible. The entire vessel wall area was automatically divided into ten evenly spaced segments by two contours (Fig. 1). Measurement values, including average outer diameter and maximum outer diameter, average wall thickness and maximum wall thickness (i.e., mean and maximum values of the ten distances between contour lines), contour area of internal and external lumens and the vessel wall area, and the relative signal intensity of the internal and external lumen contours, as well as the relative signal intensity of the vessel wall were automatically generated by the software. According to the above measurements, the wall thickness index, remodeling index, and relative signal intensity after vessel wall enhancement (ER) were obtained. When IMH was found in the dissection, the relative signal intensity of IMH and stenosis percentage were measured and calculated using the following equations: Wall thickness index = vessel wall area/external lumen contour area; Remodeling index = external lumen contour area/normal external lumen contour area; ER = SI_postBBMR_/SI_preBBMR_; SI_postBBMR_ and SI_preBBMR_ refer to the normalization of the relative signal intensity of the vessel wall after plain scan and enhancement by comparing them to the relative signal intensity of the adjacent normal vessel wall [19].

### Statistical analysis

All statistical analyses were performed using SPSS 18.0 software (IBM, Armonk, NY). Data of continuous variables are presented as mean ± standard deviation. Categorical data are presented as frequency (percentage). Fisher’s exact test was used to compare the detection rate of MRI and HRMR-VWI signs in different stages. The Kruskal–Wallis test and subsequent pairwise comparisons were performed to analyze the differences in the quantitative indicators of different stages. For all statistical analyses, *P* < 0.05 was considered to indicate a statistically significant difference.

## Results

### Clinical characteristics

A total of 38 dissecting arteries were included in 34 patients, with bialteral vessels involved in 4 patients. The mean age of the patients included in this study was 49.77 years (range, 17–68 years). In this study, hypertension (35.3%) was the risk factor with the highest incidence, followed by hyperlipidemia (32.4%; see Table 1 for details).

**Table 1:** Patients’ characteristics

	Number of patients (proportion)
Sex	Male 16 (48.5)
	Female 18 (51.5)
Risk factors, N (%)	
Hypertension	12 (35.3)
Hyperlipidemia	11 (32.4)
Diabetes mellitus	5 (14.7)
History of trauma	3 (8.8)
Recent infection	1 (3.0)
Alcohol use	2 (6.1)
Age (years, mean)	49.77
BMI (kg/m^2^, mean)	22.55

**Fig. 2:**
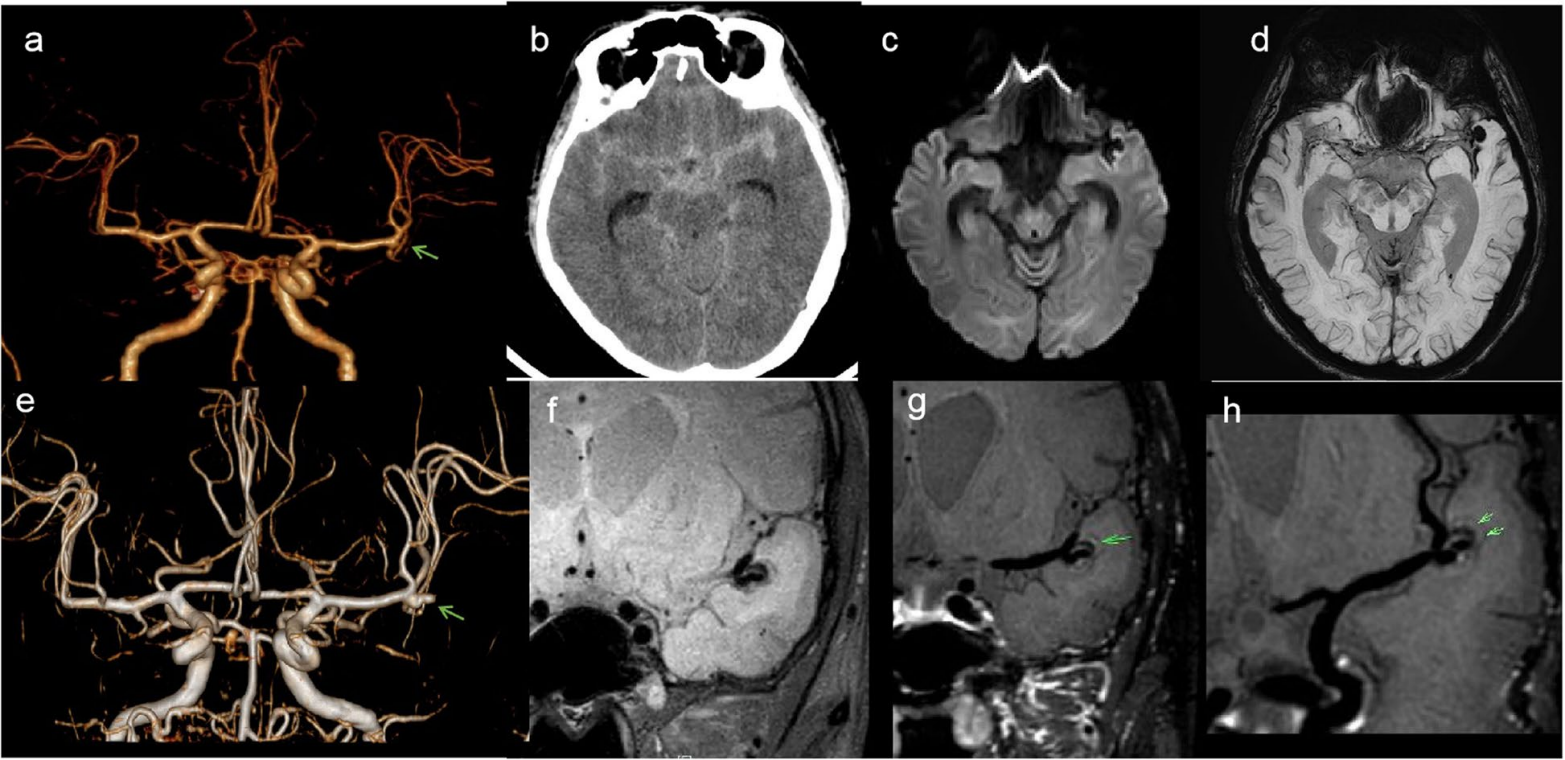
Images of a 48-year-old male patient who presented with transient loss of consciousness and acute headache for 6 h. **a** Computed tomography angiography (CTA) showed that the M1 segment was slightly swollen at the end, which was not obvious (see arrow). This patient was diagnosed as normal at the time of admission. **b** Computed tomography (CT) showing the subarachnoid hemorrhage of the patient; **c**-**d** 1 month later, diffusion-weighted imaging (DWI) and susceptibility weighted imaging (SWI) showing saccular aneurysm; **e** magnetic resonance angiography (MRA) showing an aneurysm at the M1 segment (see arrow); and **f**-**h** high-resolution magnetic resonance imaging **(**HRMR-VWI) showing aneurysmal protrusion with thickened residual wall at the end of the M1 segment and a crescent-shaped hematoma under the tunica adventitia (see arrow)

**Fig. 3:**
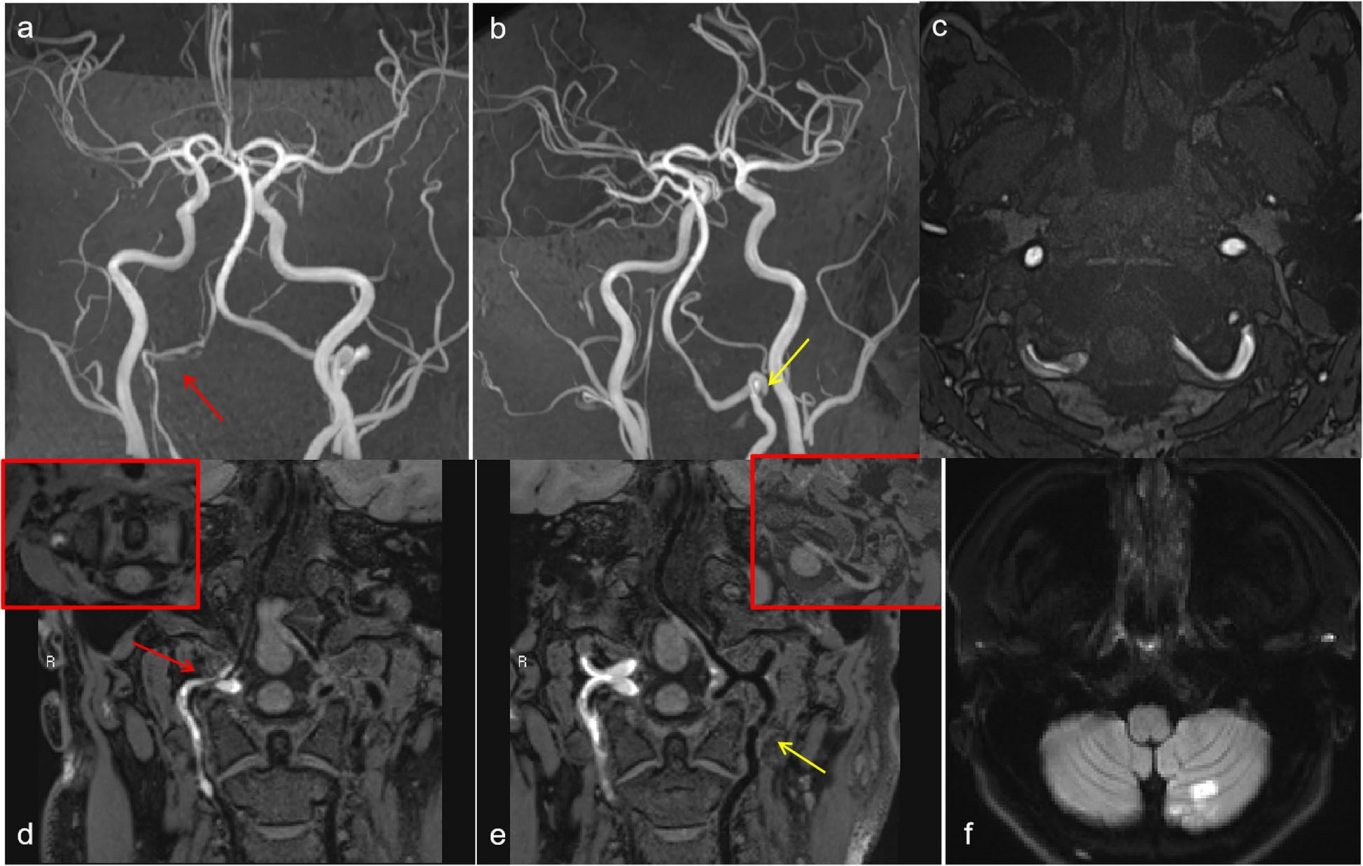
Images of a 27-year-old female patient who presented with a headache and dizziness for 8 h. **f** Acute infraction/ischemia in the left cerebellar hemisphere. **a**-**b**, **c** MRA revealed V2–V4 tapered stenosis (red arrows), and regularity and local aneurysmal dilatation of the V3 segment vessel wall of the left vertebral artery. **d** HRMR-VWI showing extensive IMH (red arrow) of the right V2–V4 segments, compressing and narrowing the true lumen; and **e** intimal-flap-tearing (yellow arrows) in the left V3 segment and an IMH in the left V3–V4 segments, compressing and narrowing the true lumen

**Fig. 4:**
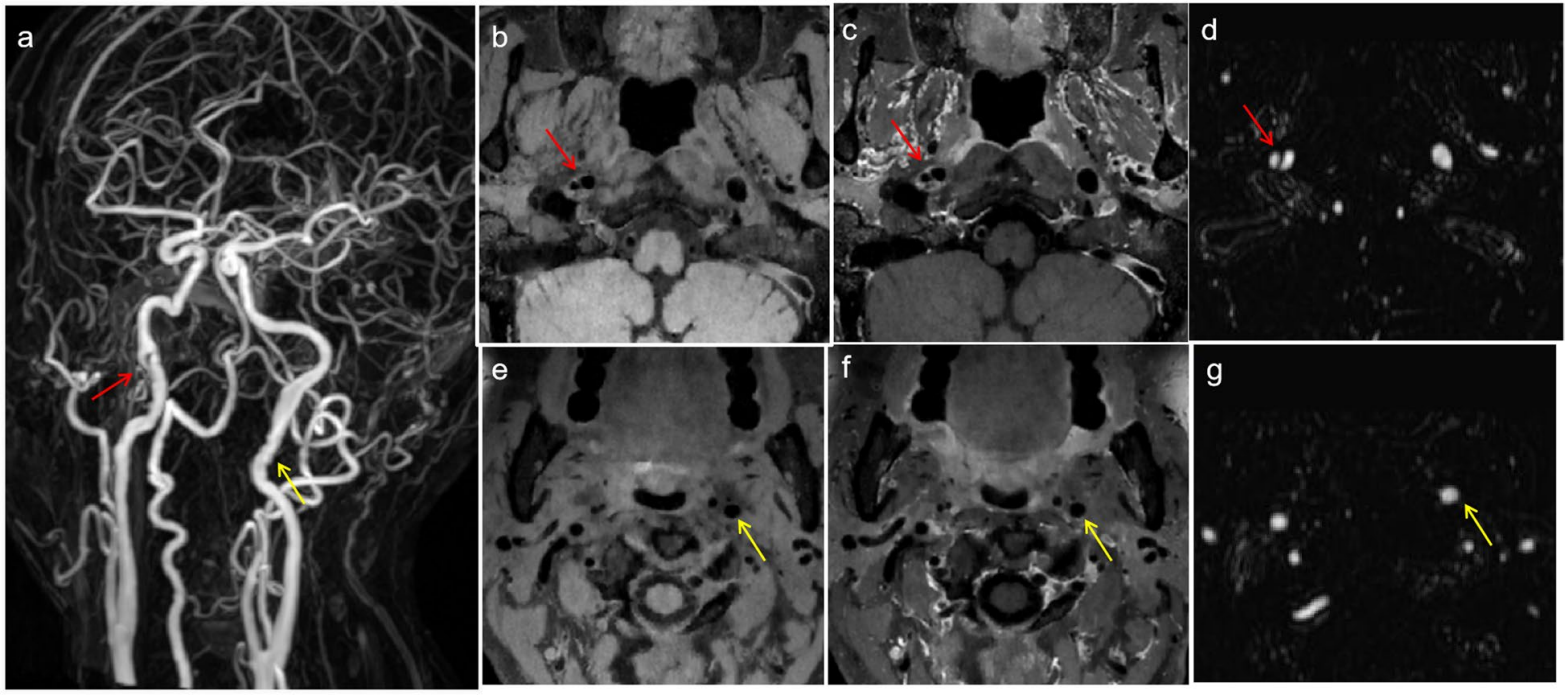
Images of a 65-year-old male patient who presented with repeated dizziness for more than 11 years and recurrence for more than 1 month. **a**, **d**, **g** MRA showing irregularity and aneurysmal dilatation of the vessel wall of the C1 segment of the right internal carotid artery. The wall of the C1 segment of the left internal carotid artery was not smooth, but no obvious abnormality was observed. **b**-**c** Imaging features of the double lumen and confined thickening of the vessel wall, showing Level 1 vessel wall enhancement; **e**-**f** the posterior wall of the lumen was thickened with confined thickening and Level 0 enhancement

**Fig. 5:**
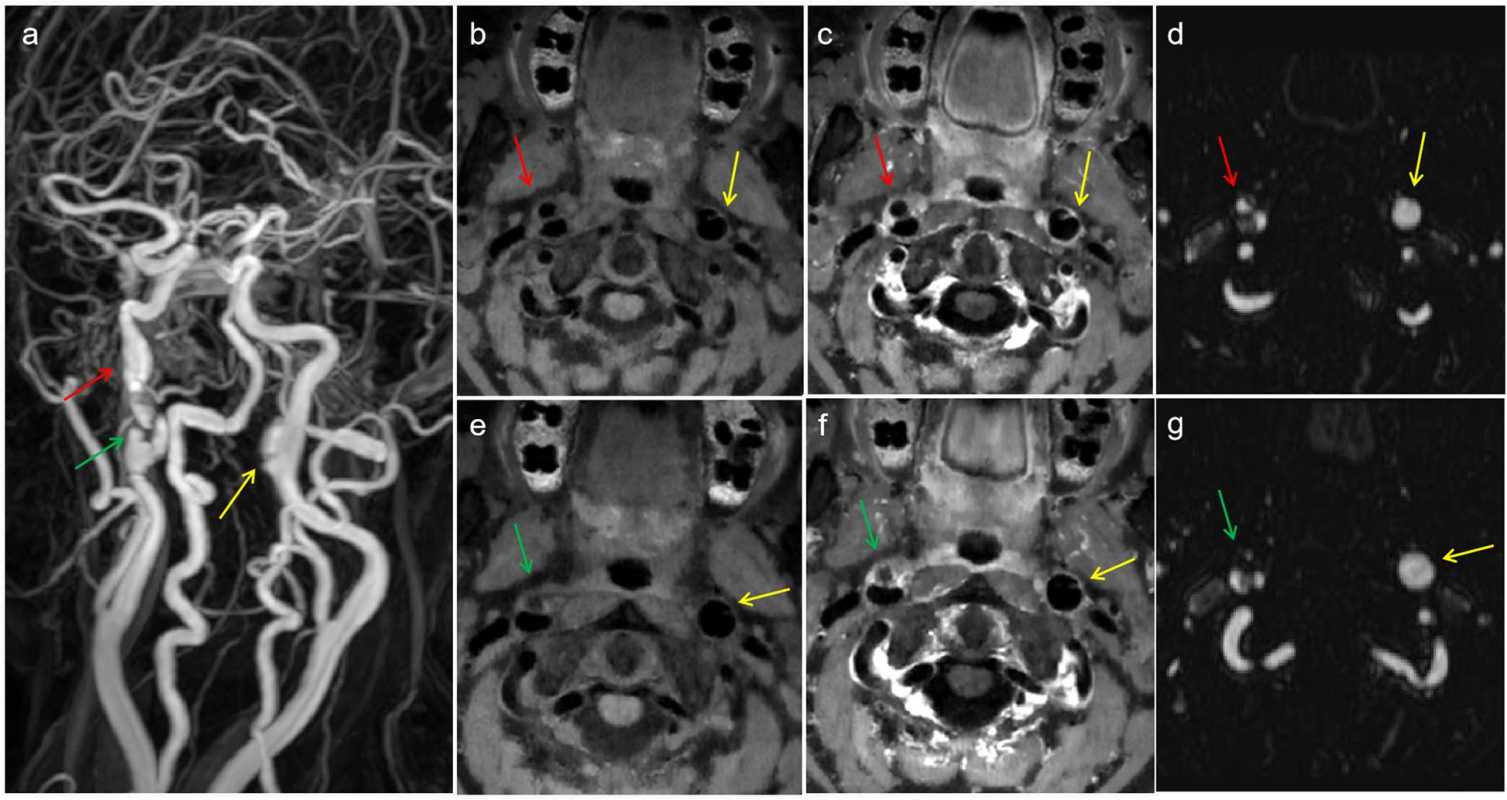
Images of a 56-year-old female patient who presented with repeated headache for more than 10 years and recurrence for 1 month. **a**, **d**, **g** MRA showing irregularity of the vessel wall and aneurysmal dilatation and protrusion of the C1 segment of the right internal carotid artery. The vessel wall of the C1 segment of the right internal carotid artery also had irregularity and aneurysmal dilatation. **b**-**c**, **e**-**f** HRMR-VWI showing imaging features of the double lumen and limited thickening of the vessel wall, as well as Level 1 vessel wall enhancement (red arrows), pseudoaneurysm formation with limited thickening of the vessel wall and Level 2 vessel wall enhancement (green arrows), and intimal-flap-tearing of the C1 segment of the left internal carotid artery (yellow arrows)

**Fig. 6:**
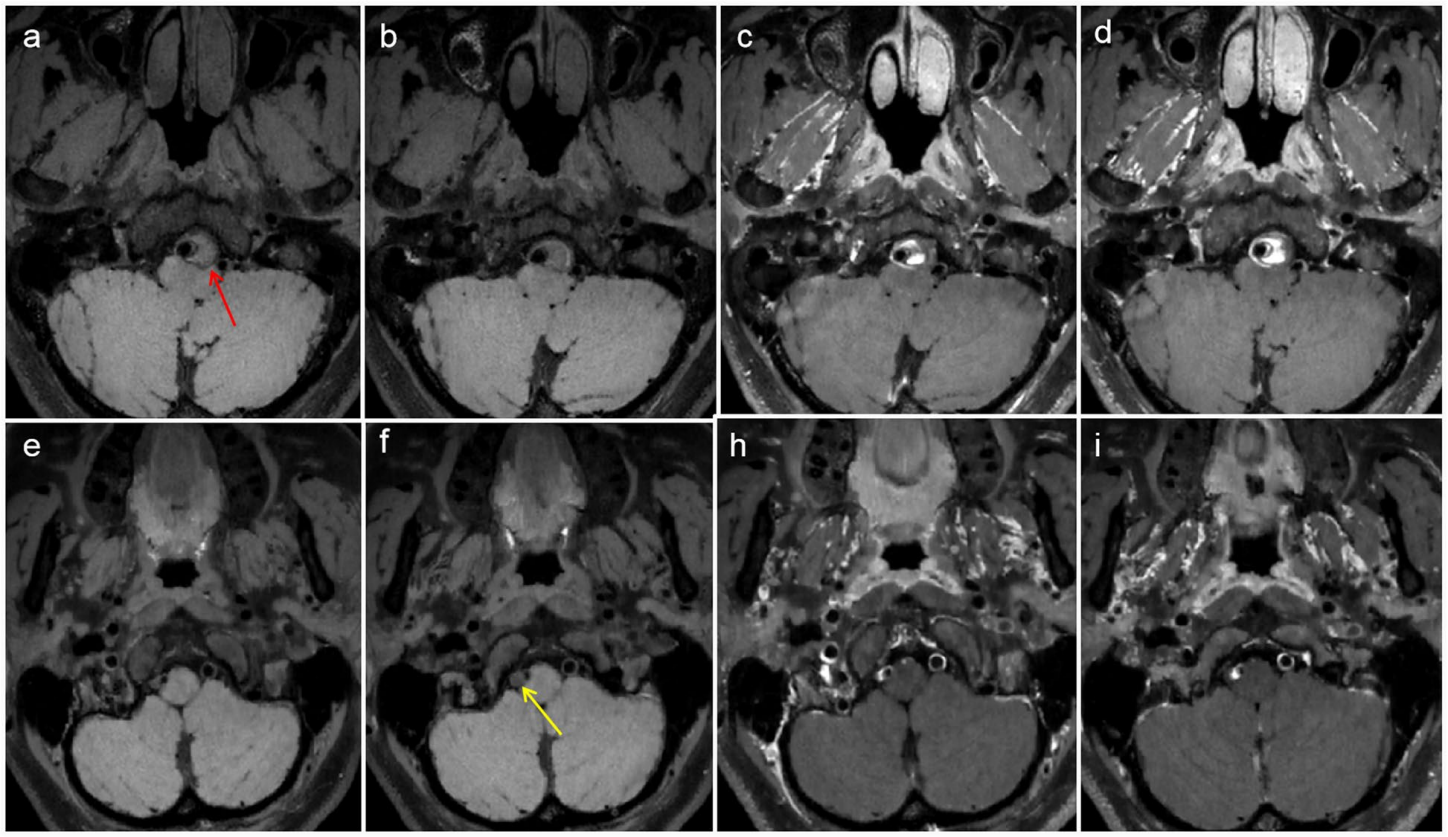
HR-MRI images of two patients. **a**-**d** HRMR-VWI taken at admission of a 55-year-old male patient who presented with repeated headache for 3 years. A thrombotic aneurysm of the V4 segment of the right vertebral artery, compressing the brain stem (mass effect) was observed. **e**-**i** HR-MRI taken during physical examination of a 43-year-old female patient with a history of hypertension showed a thrombotic aneurysm of the V4 segment of the right vertebral artery, and the compressed vessel was slightly narrowed

According to the vascular segments involved in the lesions, 38 diseased blood vessels were divided into the middle cerebral artery segment (4 vessels), the intracranial vertebral artery segment (14 vessels), and the extradural segment of the internal carotid artery (20 vessels). According to the disease stage, there were 8 diseased blood vessels in the acute stage, 8 diseased blood vessels in the subacute stage, and 20 diseased blood vessels in the chronic stage. Patients in the chronic stage were divided into the recovered group (*n* = 8) and the incompletely recovered group (*n* = 12). As shown in Table 2, four patients with dissection of the middle cerebral artery segment were hospitalized due to symptoms of subarachnoid hemorrhage; three of them were negative in the initial computed tomography angiography (CTA)/ DSA and were found to have a dissecting aneurysm in the subacute stage by HRMR-VWI (Fig. 2). The most common symptom of the cases involving intracranial vertebral artery segment was infarction/ischemia (Fig. 3). The patients with dissection in the extradural internal carotid artery segment were mainly asymptomatic and were hospitalized due to stroke or other symptoms in nonpathological vessel segments. MRA and HRMR-VWI revealed that some of them were follow-up examinations of past confirmed cases of dissection, and most of the patients were in the chronic stage (Figs. 4, 5 and 6).

**Table 2:** The different clinical characteristics of CCAD according to vessel segments

Different vessel segments	Symptoms				Stages		
	subarachnoid hemorrhage(4)	cerebral ischemi(12)	isolated headache and mass effects(4)	no symptom(18)	acute stage(8)	subacute stage(8)	chronic stage(22)
Middle cerebral artery (4)	4(100.0)	0(0.0)	0(0.0)	0(0.0)	1(25.0)	3(75.0)	0(0.0)
Intradural segment of the vertebral artery (14)	0(0)	9(64.3)	2(14.3)	3(21.4)	6(42.9)	3(21.4)	5(35.7)
Extradural segment of the carotid artery (20)	0(0)	3(15.0)	2(10.0)	15(75.0)	1(5.0)	2(10.0)	17(85.0)

### MRA and MRMR-VWI manifestations of CCAD at different stages

Table 3 shows the detection rates of CCAD signs using MRA and HRMR-VWI. In the acute stage of CCAD, the detection rates of conical stenosis or occlusion by MRA and the detection rates of IMH and Grade II enhancement of vessel wall by HRMR-VWI were significantly higher than those in the chronic stage of CCAD (*P* = 0.001/0.000/0.000). MRA did not show significantly different detection rates of the typical dissection signs of CCAD between different stages (*P* = 0.902). In contrast, HRMR-VWI detected the typical dissection signs in all of the CCAD patients, showing significant differences between acute, subacute, and chronic stages of CCAD (*P* = 0.016).

**Table 3:** MRA and HRMRI findings of CCAD at different stages

Differ-ent stages	MRA findings				The typical sign detectio-n of CCAD	HR-MRI findings							The typical sign detection of CCAD
	intimal flap or double lumen	irregular vessel wall directio-n	aneurys-mal dilatation or fusiform dilatation	tapered stenosis or occlusi-on		intimal flap	double lumen	aneurys-mal protrusi-on	IMH	grading of vessel wall enhancement			
										Level 0	Level 1	Level 2	
Acute stage (5)	1 (12.5)	5 (62.5)	3 (37.5)	6 (75.0)	3 (37.5)	2 (25.0)	3 (37.5)	1 (12.5)	7 (87.5)	0 (0.0)	0 (0.0)	8(100.0)	8(100.0)
Subac-ute stage (7)	1 (12.5)	6(75.0)	4 (50.0)	3 (37.5)	4 (50.0)	5 (62.5)	4 (50.0)	4 (50.0)	5 (62.5)	0 (0.0)	3 (42.9)	5 (62.5)	8 (100.0)
Chroni-c stage (22)	10 (45.5)	13 (59.1)	12 (54.5)	2 (9.1)	12 (54.5)	11 (50.0)	10 (45.5)	13 (59.1)	2 (9.1)	8 (36.4)	12 (54.5)	2 (9.1)	13 (59.1)
*p* value	0.180	0.896	0.902	0.001	0.902	0.368	1.000	0.095	0.000	/	/	0.000	0.016

### Comparison of typical dissection signs detected by MRA and HRMR-VWI

Table 4 shows the detection rates of the typical dissection signs of CCAD using MRA and HRMR-VWI. HRMR-VWI was significantly better in detecting intimal flap/double lumen, mainly in the early stage of CCAD, compared with MRA (*P* = 0.012). However, the two imaging methods showed no significant differences in detecting the typical dissection signs in the chronic stage of CCAD (*P* = 1.000) or in detecting tapered stenosis or occlusion–intramural hematoma, aneurysmal dilation, or protrusion.

**Table 4:** Comparison of typical dissection signs detected by MRA and HRMR-VWI

The typical sign detection of artery dissection	MRA	HRMR-VWI	Χ^2^ value	*p* value
Early stage(acute and subacute stage)				
Tapered stenosis or occlusion or IMH	9(56.3)	12(75.0)	1.247	0.264
Intimal flap or double lumen	3(18.8)	10(62.5)	6.348	0.012
Aneurysmal dilatation or protrusion	7(43.8)	8(50.0)	0.125	0.723
Chronic stage				
Tapered stenosis or occlusion or IMH	2(9.1)	2(9.1)	/	1.000
Intimal flap or double lumen	10(45.5)	11(50.0)	0.000	1.000
Aneurysmal dilatation or protrusion	12(54.5)	13(59.1)	0.093	0.761
At all stages				
Tapered stenosis or occlusion or IMH	10(26.3)	13(34.2)	0.561	0.454
Intimal flap or double lumen	12(31.6)	21(55.3)	4.338	0.037
Aneurysmal dilatation or protrusion	19(50.0)	21(55.3)	0.211	0.646

### Quantitative parameters of vessel walls of CCAD at different stages

**Fig. 7:**
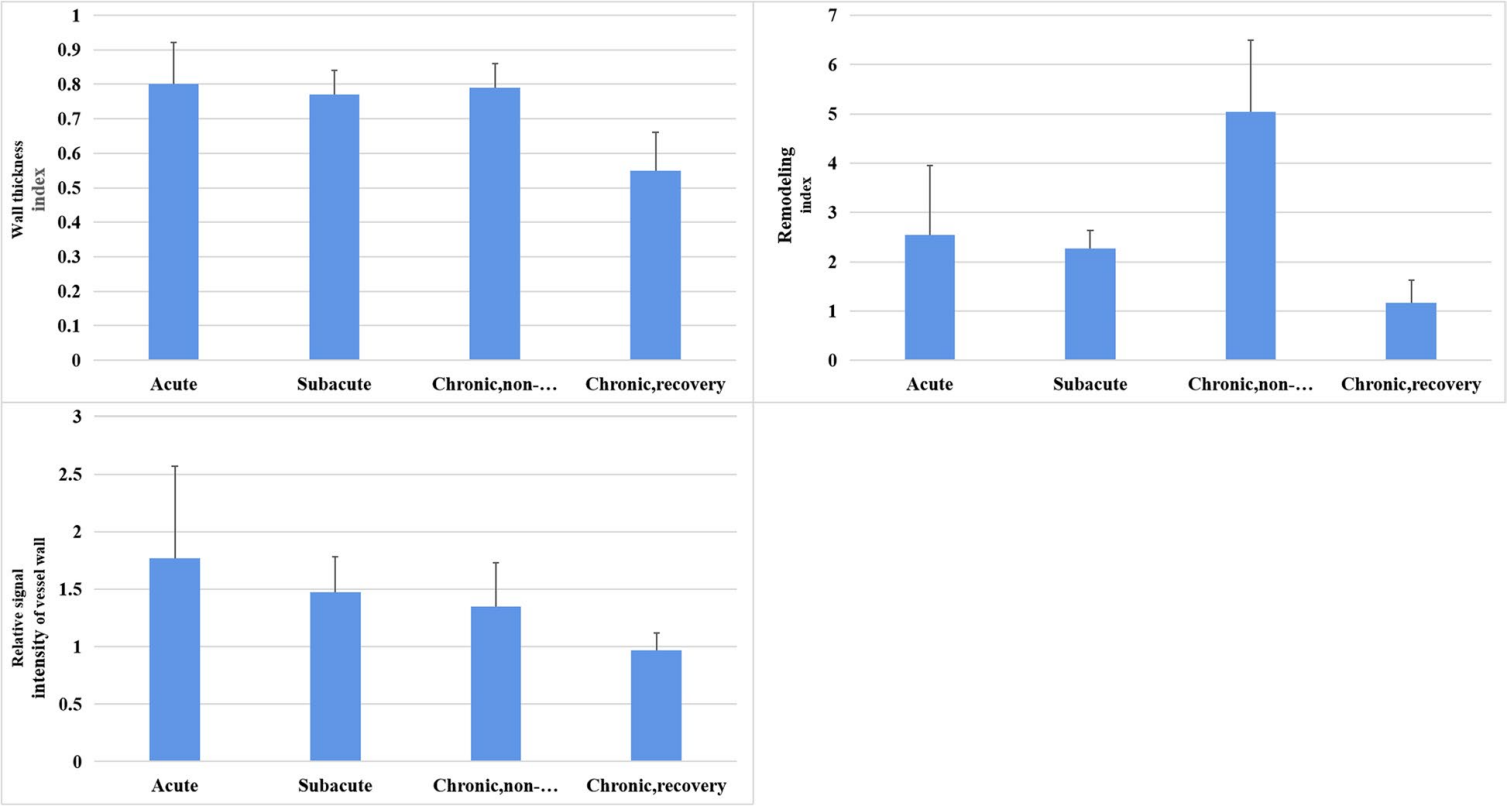
Quantitative parameters of vessel walls of CCAD at different stages

**Fig. 8:**
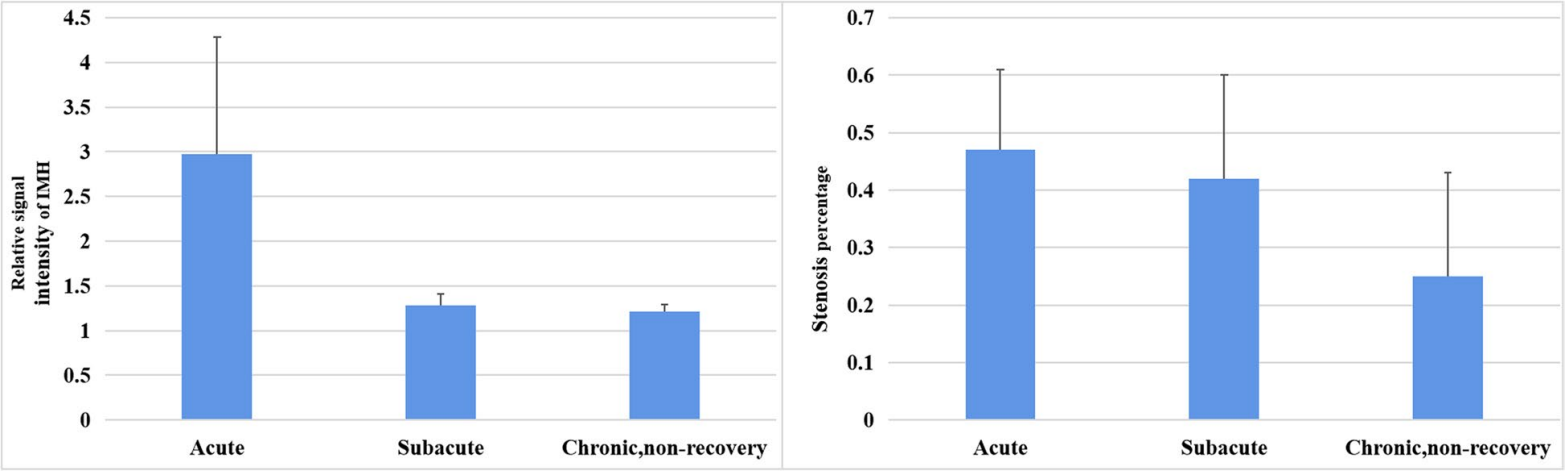
Quantitative parameters of vessel walls of CCAD at different stages

The values of wall thickness, relative signal intensity of vessel wall enhancement, relative signal intensity of IMH, and percentage of stenosis in CCAD decreased from acute to subacute and then to chronic stages. Each quantitative parameter in patients with CCAD in the early stages (i.e., acute and subacute stages) was significantly different from that in patients with CCAD in the recovered group at chronic stage (*P* < 0.05, Table 5; Figs. 7 and 8). Wall thickness and relative signal intensity of vessel wall enhancement in patients with CCAD in the early stages were not significantly different from those in patients with CCAD in the incompletely recovered group at chronic stage (*P* > 0.05, Table 5; Figs. 7 and 8).

**Table 5:** Quantitative parameters of vessel walls in different vessel segments and stages

Different stages	Wall thickness index	Remodeling index	Relative signal intensity of vessel wall	Relative signal intensity of IMH	Stenosis percentage
Acute stage	0.80 ± 0.12	2.54 ± 1.41	1.77 ± 0.80	2.97 ± 1.31	0.47 ± 0.14
Subacute stage	0.77 ± 0.07	2.27 ± 0.37	1.47 ± 0.31	1.28 ± 0.13	0.42 ± 0.18
Chronic stage, non-recovery	0.79 ± 0.07	5.04 ± 1.45	1.35 ± 0.38	1.21 ± 0.08	0.25 ± 0.18
Chronic stage, recovery	0.55 ± 0.11	1.17 ± 0.46	0.97 ± 0.15		
Acute-chronic, recovery	0.000	0.000	0.000		
Subacute-chronic,recovery	0.002	0.000	0.001		
Acute-chronic, non-recovery	1.000	0.000	0.061	0.000	0.000
Subacute-chronic,non-recovery	1.000	0.008	1.000	1.000	0.004

## Discussion

This study used MRA and HRMR-VWI to evaluate changes in the lumen and the vessel wall of patients with CCAD at different stages and compare the detection of typical dissection signs. Our results showed that HRMR-VWI had unique advantages in the early diagnosis of CCAD and detected the typical signs of dissection, especially the intimal flap/double lumen, in almost all of the patients at the early stage of CCAD. Although the diagnostic performance of MRA was not as good as that of HRMR-VWI, MRA still detected the typical signs of dissection in more than half of the patients; moreover, compared with HRMR-VWI, MRA showed no significant differences in the detection of typical signs of dissection at the chronic stage. HRMR-VWI showed higher detection rates of the typical dissection signs at the early stages (acute and subacute stages) than at the chronic stage of CCAD. The changes of different stages in intramural hematoma, relative signal intensity of blood vessel wall, and quantitative indicators of wall thickness detected by HRMR-VWI effectively guided the disease course staging and of CCAD, whereas MRA failed to reveal any differences in the typical dissection signs of CCAD at different stages.

### Analyses of different clinical manifestations of CCAD

The intradural segment of the cervical internal carotid artery began at the oblique process (C6) of the carotid artery, and the intradural segment of the vertebral artery began at the V4 segment. Because the intradural segment of the intracranial artery was mainly surrounded by cerebrospinal fluid, the supporting tissues surrounding the vessel segment were relatively weak and had different histological characteristics from the extradural segment of the carotid artery; the internal elastic lamina was relatively thick and dense, with few elastic fibers in the tunica media, less tissues, and lack of elastic fibers in the tunica adventitia. Thus, the internal elastic lamina was more prone to sub-adventitial dissection and subsequent subarachnoid hemorrhage [1, 20]. In this study, patients with dissections of the vessel segments of the middle cerebral artery were hospitalized due to subarachnoid hemorrhage. There were three negative cases detected in the initial CTA/DSA. However, these patients were detected with a dissecting aneurysm and surrounding sub-adventitial hemorrhage by HR-MRI in the subacute stage. As sub-adventitial dissection is difficult to detect and diagnose in luminal imaging, it is easy to miss the diagnosis in the acute stage. Thus, HR-MRI should be used to exclude the possibility of dissecting aneurysm when the cause of subarachnoid hemorrhage cannot be determined by intraluminal angiography.

The main symptom of dissection that occurred in the intracranial segment of the vertebral artery was infarction and ischemia, mostly and simultaneously involving the V3–V4 segments. Given that the greatest reduction in the elastic fibers of the tunica media and the external elastic lamina of the vertebral artery is in the 0.5 cm regions before and after the intradural segment, and that the blood flow changes immediately at the proximal and distal ends of the dissection, the intracranial segment of the vertebral artery often involves the V3–V4 segments [1]. In addition, more perforating vessels in the posterior circulation are prone to infarction in perforating arteries [21]. In this study, the clinical manifestations of patients in the internal carotid artery extradural segment group were mainly asymptomatic, and most patients were in the chronic stage when undergoing HR-MRI. In contrast to ICAD, extracranial artery dissection (ECAD) was not a common cause of infarction, and greater than 50% of the patients did not prompt the diagnosis of ECAD. Cerebral infarction accounted for 40–60% in ECAD, while transient ischemic attack (TIA) accounted for 20–30%. Moreover, the mechanism of infarction generally originated from embolism, which frequently led to cortical or subcortical infarction, challenging the early clinical diagnosis [2, 22].

### Evaluation of MRA and HRMR-VWI performances in the detection of typical dissection signs in CCAD

Given the maturation, short imaging duration, and the lack of requirement of a contrast agent, MRA is often used as a routine examination method for the initial evaluation of stroke. A comparative study of MRI/MRA and DSA in CCAD diagnosis showed that MRI/MRA was no less accurate than DSA in detecting the typical signs of dissection, and MRI also detected IMH that DSA could not [23]. In this study, we compared the detection rates of the typical signs of dissection between MRA and HRMR-VWI, and showed that the typical signs of dissection were detected in more than half of the patients. Among them, tapered stenosis or occlusion was the most common sign at the early stages of CCAD. However, MRA’s detection of intimal flap and dual lumen was not as good as that of HRMR-VWI. In addition, the lumen imaging features are all indirect signs of CCAD. Using lumen features to diagnose CCAD relies too much on the experience of radiologists and physicians and is highly subjective, and may miss patients with nonspecific tapered stenosis or occlusion sign [6].

HRMR-VWI could directly describe and evaluate the changes in the vessel wall to characterize the direct signs of dissection, showing more advantages than MRA in CCAD diagnosis. In this study, application of HRMR-VWI also revealed the typical signs of dissection in almost all of the CCAD patients, especially the intimal flap/double lumen, indicating the unique advantage of HRMR-VWI. We also showed that MRA failed to reveal any abnormal findings in some cases of CCAD or revealed only nonspecific tapered stenosis or occlusion. In contrast, HRMR-VWI revealed dissecting aneurysm with thrombosis or intramural hematoma in those cases because CCAD had often undergone positive remodeling, and CCAD without stenosis or intratumoral thrombosis was not easy to detect only by lumen imaging. However, HRMR-VWI was able to more sensitively detect intramural hematoma, which was not revealed by MRA or DSA [23, 24]. HRMR-VWI could also clarify the extent of dissection and provide information for differential diagnosis of CCAD from other vascular lesions [25, 26].

### Evaluation of quantitative imaging indicators of CCAD acquired by HRMR-VWI

In this study, MRA failed to reveal any differences in the typical signs of dissection at different stages of CCAD. Since lumen imaging only shows the results of disease changes but does not describe the state of the disease changes, it cannot be used effectively in staging the course of CCAD. Unlike MRA, HRMR-VWI reveals the changes during the CCAD healing process. Park et al. compared the imaging manifestations of CCAD between different stages and identified intimal flap, double lumen, intramural hematoma, and vascular ectasia at the early stage of the disease. These signs were not pronounced at the chronic stage of CCAD (60 days after the disease onset) [27]. These signs are consistent with our findings. Namely, the detection rates of the typical signs of dissection by HRMR-VWI at the acute and subacute stages were higher than those at the chronic stage, and the detection rate of IMH was the highest, reaching 87.5%. Quantitative assessment showed that the relative signal intensity of IMH decreased from the acute stage to the chronic stage, which was consistent with the signal changes in parenchymal hematoma. Compared with parenchymal hematoma, the absorption of IMH in dissection was relatively slow, which might be related to the different degradation environment of the hematoma or repeated bleeding inside the hematoma [28]. Therefore, the change in the relative signal intensity of IMH could be used as a quantitative indicator to assess the staging of CCAD by HRMR-VWI.

Vessel wall enhancement is common in CCAD at the acute stage, and the relative signal intensity of vessel wall enhancement decreases as CCAD progresses from the acute stage to the chronic stage. This may be related to the initiation of repair and reconstruction of the affected arteries at the early stage of the disease, the infiltration by inflammatory cells, the formation of new blood vessels, and the presence of nourishing vascular wall that causes vascular wall enhancement. Absence of histological signs of inflammation in the vessel wall refers to the lack of vascular wall enhancement [12, 29]. Hashimoto et al. conducted a retrospective analysis of the degree of vascular wall enhancement in 49 patients with unruptured ICAD and showed that the contrast enhancement ratio of vessel wall to the pituitary stalk in patients with progressive ICAD was significantly higher than that in patients with improved and stable ICAD. Quantitative analysis of contrast-enhanced HRMR-VWI predicted the instability in unruptured ICADs [30]. Therefore, quantification of changes in vessel wall enhancement by HRMR-VWI provided important information for CCAD staging and risk stratification.

Quantitative evaluation in this study showed that in addition to intramural hematoma and relative signal intensity of vessel wall, the wall thickness also decreased as CCAD progressed from the acute stage to the chronic stage. A previous study conducted histological examination of vascular tissue specimens from patients with CCAD at different time intervals and showed compensatory hyperplasia of the intima and injury repair [13]. Thus, small changes in the vessel wall due to compensatory repair during the healing process can be characterized by HRMR-VWI [4]. In this study, the analysis of quantitative indicators of HRMR-VWI showed thatthere was no difference in wall thickness between patients at the acute stage and those in the incompletely recovered group at the chronic stage; in contrast, it revealed the significant difference in wall thickness between patients at the acute stage and those in the recovered group at the chronic stage. These results indicated that the change in wall thickness as detected by HMRM-VWI may be a potential marker for the prediction of CCAD progression.

This study has several limitations. First, it was a retrospective study involving a small number of patients and may have had sample selection bias, which limited us from drawing a general conclusion. Second, this study mainly used TOF-MRA for luminal imaging, which is prone to show blood flow-related artifacts and has image distortion, thus having limited value in lumen evaluation. Third, this study only included a small amount of interindividual data. CCAD is a dynamic repair process, and the characteristics of different vessel segments and at different disease stages are affected by multiple factors, including risk factors, symptoms, and individual variations, which may have affected our findings as well. Therefore, additional follow-ups and prospective studies are necessary to determine the predictive power of these imaging features.

## Conclusions

As the only noninvasive imaging technology, HRMR-VWI displays the structure of the vessel wall in vivo, showing not only excellent performance in the early diagnosis of CCAD, but also describing the changes in the qualitative and quantitative characteristics of vessel wall. It also helps to guide the disease diagnosis, course staging and treatment of CCAD. Although the diagnostic performance of MRA is not as good as that of HRMR-VWI, in our study, it detected the typical signs of dissection in more than half of the CCAD patients, and MRA showed no significant differences compared with HRMR-VWI in CCAD patients at the chronic stage. Given the many advantages of MRA, including a mature technology, short imaging duration, and not requiring a contrast agent, MRA should be the first choice of method for routine examination in evaluating CCAD, especially at the chronic stage of CCAD.

## Data Availability

All data generated or analysed during this study are included in this published article.

## Abbreviations

MRA: Magnetic resonance angiography
HRMR-VWI: High resolution magnetic resonance vessel wall imaging
DSA: Digital subtraction angiography
CCAD: Cervicocranial artery dissection
ICAD: Intracranial artery dissection
IMH: Intramural hematoma
